# Preliminary investigation on predicting postoperative glioma recurrences based on a multiparametric radiomics model

**DOI:** 10.3389/fonc.2025.1592881

**Published:** 2025-06-19

**Authors:** Fang-Xiong Fu, Guo Li, Lan Hong, Wang-Sheng Chen

**Affiliations:** ^1^ Department of Radiology, Hainan General Hospital (Hainan Medical University Hainan Hospital), Haikou, China; ^2^ Department of Radiology, Shenzhen Longhua District Central Hospital, Shenzhen, China; ^3^ Department of Gynecology, Hainan General Hospital (Hainan Medical University Hainan Hospital), Haikou, China

**Keywords:** glioma, relapse, radiomics, magnetic resonance imaging, individualized clinical treatment

## Abstract

**Background:**

The early prediction of postoperative recurrence and high recurrence area of gliomas is important for individualized clinical treatment. This study aimed to evaluate the performance of a magnetic resonance imaging (MRI)-based multiparametric radiomics model for the early prediction of postoperative recurrences.

**Methods:**

The data from 60 patients who met the inclusion criteria between 2000 and 2021 were collected in this study. Radiological features were extracted from the T1-weighted imaging (T1WI) and T2WI/fluid-attenuated inversion recovery sequence images. The multiparametric model was composed of two classifiers, the support vector machine and the logistic regression (LR), and it was used for training and prediction. The highest scoring classifiers and sequences were screened out according to the area under the curve (AUC) and accuracy.

**Results:**

For predicting the postoperative recurrences and high recurrence areas of gliomas, the performance of the LR classifier was most stable, and the multiparametric model based on clinical information, basic imaging, and radiomics had the best performance (AUC: 0.99; Accuracy: 0.96).

**Conclusion:**

The MRI-based multiparametric radiomics method provided a non-invasive, stable, and relatively accurate method for the early prediction of postoperative recurrences, which has guiding importance for individualized clinical treatment.

## 1 Background

Glioma is the most common primary tumor of the human brain, approximately 80% of which are malignant (1). High-grade gliomas, especially glioblastomas, are the most common and aggressive subtype of glioma (2, 3). Despite the choice of treatment comprising maximal safe resection combined with standard therapeutic regimens of radiotherapy and/or chemotherapy (4), most patients have a poor prognosis with a median survival of 12–15 months, which is mainly correlated with a recurrence of the tumor/residual tumor (5). Most patients may relapse in the short term, and approximately 90% of the recurrence sites are within the range of radiotherapy (6). Recurrent tumors destroy the blood–brain barrier, and the nodules with abnormal enhancements on contrast-enhanced T1-weighted imaging (CET1WI) are the targets for therapy after recurrences. Therefore, predicting the patients who will have a recurrence and their recurrence sites early may lead to early and active treatment, thereby reducing the risk of recurrence and improving their prognoses.

Based on the 2016 update of the World Health Organization (WHO) classification of tumors of the central nervous system, the 5th edition published in 2021 advances the integration of molecular diagnostics with a histopathological assessment of brain tumors, including dividing the previously designated glioblastoma entities into isocitrate dehydrogenase (IDH)-wild-type glioblastoma and IDH-mutant glioblastoma (7). A recurrence is correlated with the tumor’s spatial heterogeneity, invasiveness, and vascular proliferation. The high expression of antigen KI-67 (Ki-67) is correlated with the invasiveness, as well as the cellular and vascular proliferation of the tumor (8). It has been shown that a patient’s age, the extent of resection, and expressions of Ki-67 and IDH are correlated with poor postoperative prognoses in gliomas (9). By combining positron emission tomography (PET) and magnetic resonance imaging (MRI) with available tumor-related molecular understanding, a wealth of information about the relevant tumor tissue may be captured to assist in the diagnosis and treatment of gliomas (10). Due to the heterogeneity of gliomas, many tumor microenvironments may not be observed by the naked eye and are limited by multiple factors, such as the resolution, evaluation parameters, and doctor’s experience. What a doctor can see with solely their eyes is still unable to meet the requirements of any clinical precision treatment when evaluating the recurrence of a tumor.

Radiomics refers to the mining, high-throughput extraction of features from medical images that characterize the underlying pathophysiology of tumors (11). With the emerging technologies such as shape, size, and texture features (12, 13), the clinical features and molecular markers may be taken as additional input features and combined with radiomics (technologies defined as high-throughput biochemical assays that measure comprehensively and simultaneously molecules of the same type from a biological sample) features to build predictive models. Therefore, quantitative assessment of imaging and the development of imaging biomarkers using radiomics may assist in understanding the biology of tumors. Many studies (14, 15) have shown that radiography combined with radiomics technology has a high predictive ability in the diagnosis, grading, phenotype prediction, and treatment evaluation of tumors (16). In recent years, advances in computer technology have led to breakthroughs in radiomics-related research, which is now being increasingly applied in the detection of the invasive margins of gliomas, distinguishing the treatment-related changes from the true tumor progression, and predicting the invasiveness, risk of future recurrence, and overall survival, to achieve real-time tumor therapy monitoring (17).This study aims to evaluate the multiparametric radiomics model’s ability to predict the postoperative recurrence of gliomas based on conventional MRI sequences and attempts to identify the gross area of recurrence, which might guide the delineation of clinical surgical resections and postoperative radiotherapy regimens.

## 2 Materials and methods

### 2.1 General data

**Figure 1:**
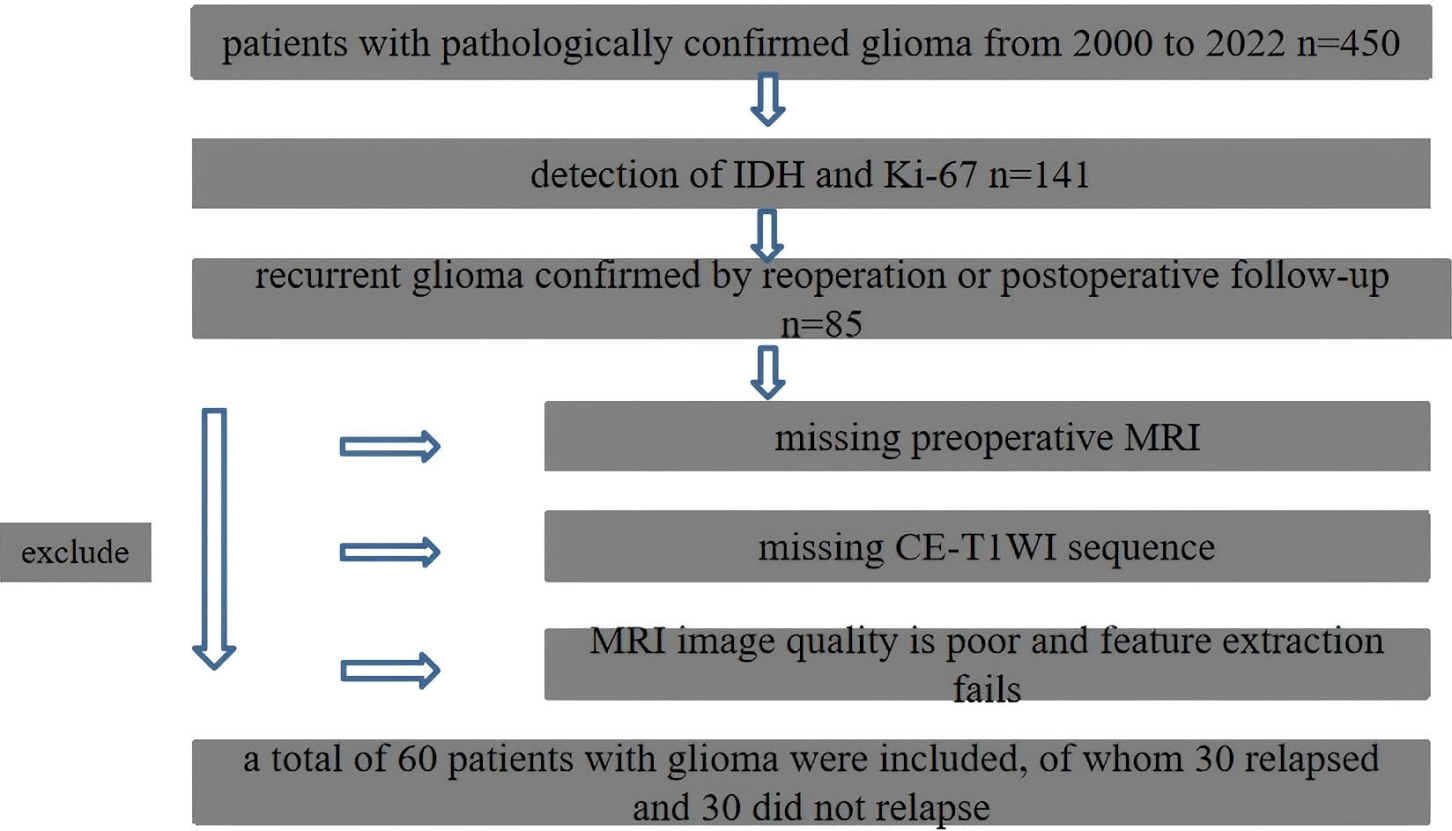
Flowchart of patient inclusion and exclusion criteria.

A retrospective cross-sectional approach was adopted in this study, and the data from 450 patients with pathologically confirmed gliomas who were treated in the Hainan Provincial People’s Hospital between February 2000 and January 2022 were collected. The inclusion criteria were as follows: (1) adult patients that had a primary glioma and had histopathological proof of a history of central nervous system malignancy; (2) patients that were tested for IDH and Ki-67 expression; (3) patients that had preoperative and postoperative axial T1WI enhancement and T2WI/FLAIR data; (4) the MRI images obtained had no artifacts affecting image observation and post-processing; (5) patients who had not received radiotherapy, chemotherapy, or other treatments before surgery; and (6) patients that had a recurrence and the existence of measurable enhanced lesions on CET1WI MRI within the 80% isodose range after concurrent chemoradiotherapy. As a result, there were 60 cases of patients included. Thirty of these patients had a recurrence, and 30 had not (**Figure 1**). The Institutional Review Board approved this study, and the requirement for written informed consent was waived.

### 2.2 Image acquisition and preprocessing

The conventional MRI images were obtained in all patients after tumor resection. The Digital Imaging and Communications in Medicine images were imported into the local computer by the picture archiving and communication system. All MRI images came from different scanners. To eliminate the impact of different scanning parameters and physiological differences among patients, the FMRIB software library (http://fsl.fmrib.ox.ac.uk/fsl/fslwiki/FSL) was adopted for registration. The image was re-sampled to a voxel size of 1 × 1 × 1 mm^3^ by the custom software using linear interpolation before feature extraction.

### 2.3 Tumor segmentation

**Figure 2:**
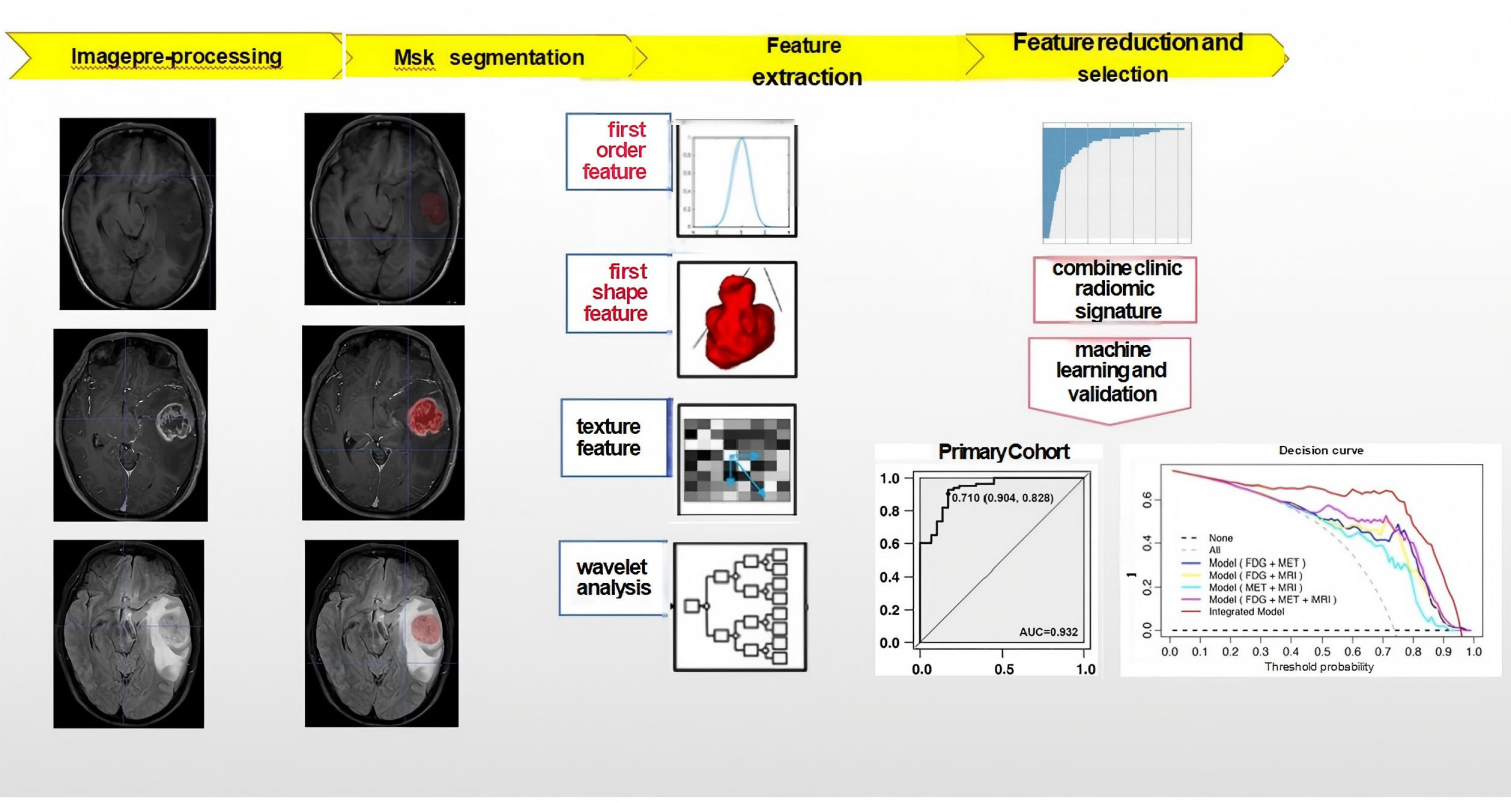
Flowchart, including image preprocessing, ROI segmentation, feature extraction, feature dimension reduction, modeling and model performance evaluation.

**Figure 3:**
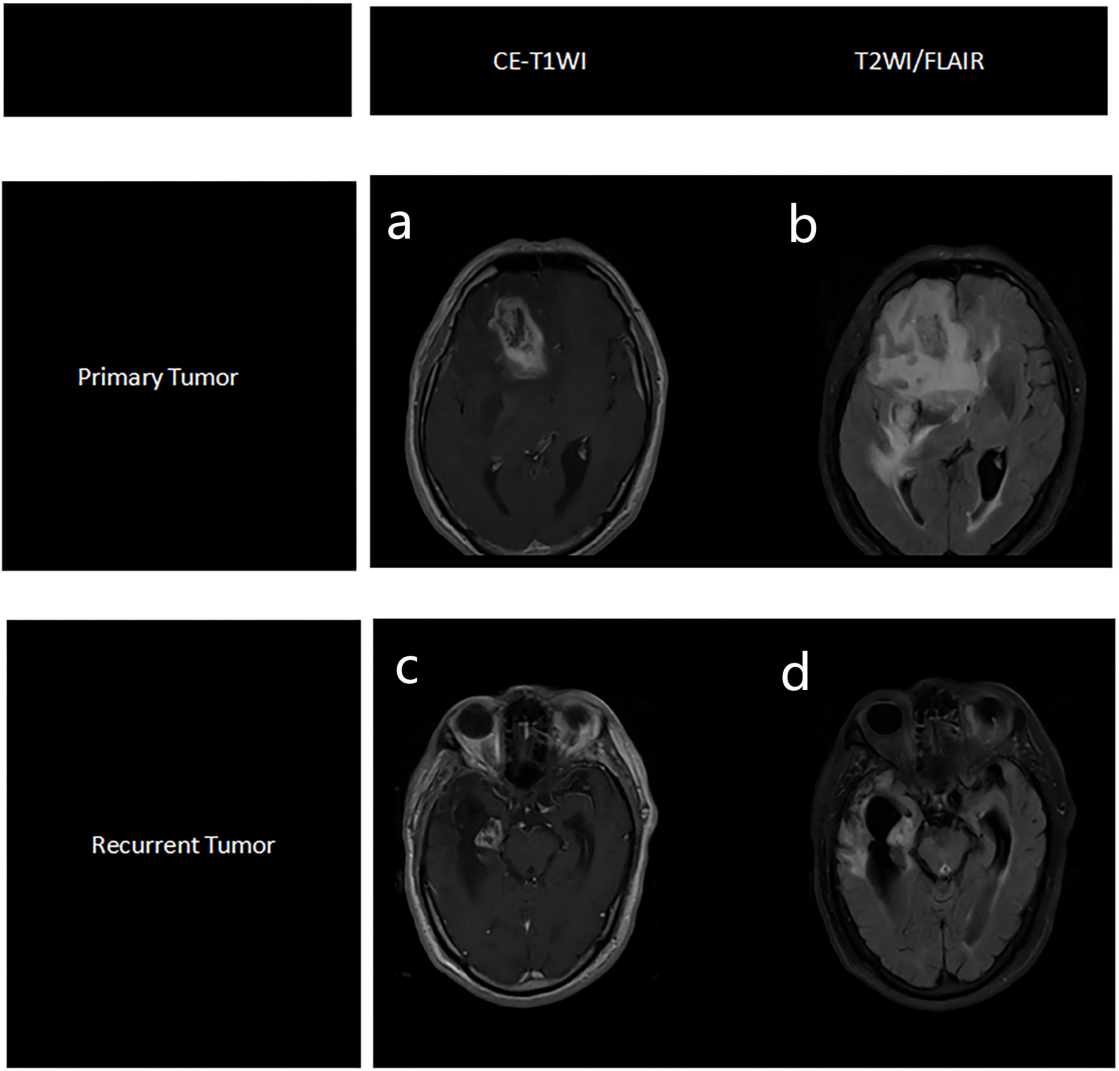
Representative MRI images of primary tumor, based on CE-T1WI sequence **(a)** and T2WI/FLAIR sequence **(b)**, and recurrent tumors, based on CE-T1WI sequence **(c)** and T2WI/FLAIR sequence **(d)**.

The enhanced lesions were manually segmented for all subjects on T1WI using the ITK-SNAP (version 3.4.0; http://www.itksnap.org) software (13). The region of interest (ROI) included the entire tumor while avoiding the blood vessels and identifiable peritumoral edemas. Finally, the mask was registered to the T2WI/fluid-attenuated inversion recovery (FLAIR) sequence. This process was conducted by a clinical radiologist with 3 years of experience and verified by a senior radiologist with 20 years of experience, who was blind to the final outcome. The analysis flow chart and typical images used in this study are shown in **Figures 2** and **3**.

### 2.4 Feature extraction and preprocessing

3D Slicer (version 5.0.2; https://www.slicer.org/) was used to extract features from each enhanced lesion and the surrounding T2WI/FLAIR high signal components, including first-order statistical features, shape features, and second-order gray level co-occurrence matrix (GLCM), gray-level run-length matrix, and gray-level size zone matrix (GLSZM). There were filters (the wavelet transforms, Laplacian of Gaussian, square root, and the log and exponent of the square root) built into these features. In order to eliminate the difference in the scale of extracted feature values, normalization and standardization were performed before feature extraction. Zscores were normalized for each feature of all patients, the mean was subtracted, and divided by SD.

### 2.5 Model construction

A total of three radiomics models were constructed, followed by feature screening and training: (i) Molecular typing prediction model to predict the expression status of molecules, including the five characteristics of gender, age at diagnosis, radiomics characteristics, location of lesions (frontal lobe, temporal lobe, parietal lobe, or occipital lobe), and degree of edema (none, mild, or obvious); (ii) Recurrence prediction model based on group characteristics, and (iii) Recurrence prediction model based on radiomics and clinical characteristics, early prediction of postoperative recurrence of glioma. This combined radiomics and radiological model was validated by the receiver operator characteristic curve, calibration curve, and detrended correspondence analysis.

### 2.6 Feature screening and training

The least absolute shrinkage and selection operator (LASSO)-logistic regression was adopted to screen out the most relevant features. The SelectKBest method was adopted to screen out the ten most relevant features. The support vector machine (SVM) and logistic regression (LR) classifiers are robust machine learning classifiers that are often applied in biomedical data classification. In this work, the test set and training set are divided into test set and training set according to the ratio of 7:3, and we use SVM and LR to train the training set. At the same time, a 5-fold cross-validation scheme is applied to avoid training bias. The features with the highest frequency in all the training were determined as the most discriminative features in the training set. The model’s performance was then validated in an independent test cohort.

### 2.7 Statistical analysis

The measurement data were tested for a normal distribution, and those that satisfied the normal distribution characteristics were compared. Age was represented by mean ± standard deviation (X ± s), and an independent samples t-test was adopted to compare groups. The chi-square test was used for countable data, such as gender and molecular typing. The SPSS 25.0 software was used for data analysis.

## 3 Results

### 3.1 General data of the subjects

The difference in gender between the recurrence group and the control group was not statistically significant (X^2^ = 1.424, P = 0.233), while the difference in Ki-67 expression between the two groups was statistically significant (X^2^ = 21.592, P < 0.001). The average ages of the two groups were 45.7 ± 15.0 years and 30.0 ± 17.1 years, respectively, and the difference was statistically significant (t = 3.658, P < 0.001).

### 3.2 Feature extraction

There were 1,064 radiomics features extracted based on T1-enhanced and T2WI/FLAIR sequences, respectively. With the exclusion of some invalid information and also the LASSO screening, a total of 39 features were extracted.

### 3.3 Feature correlation

**Figure 4:**
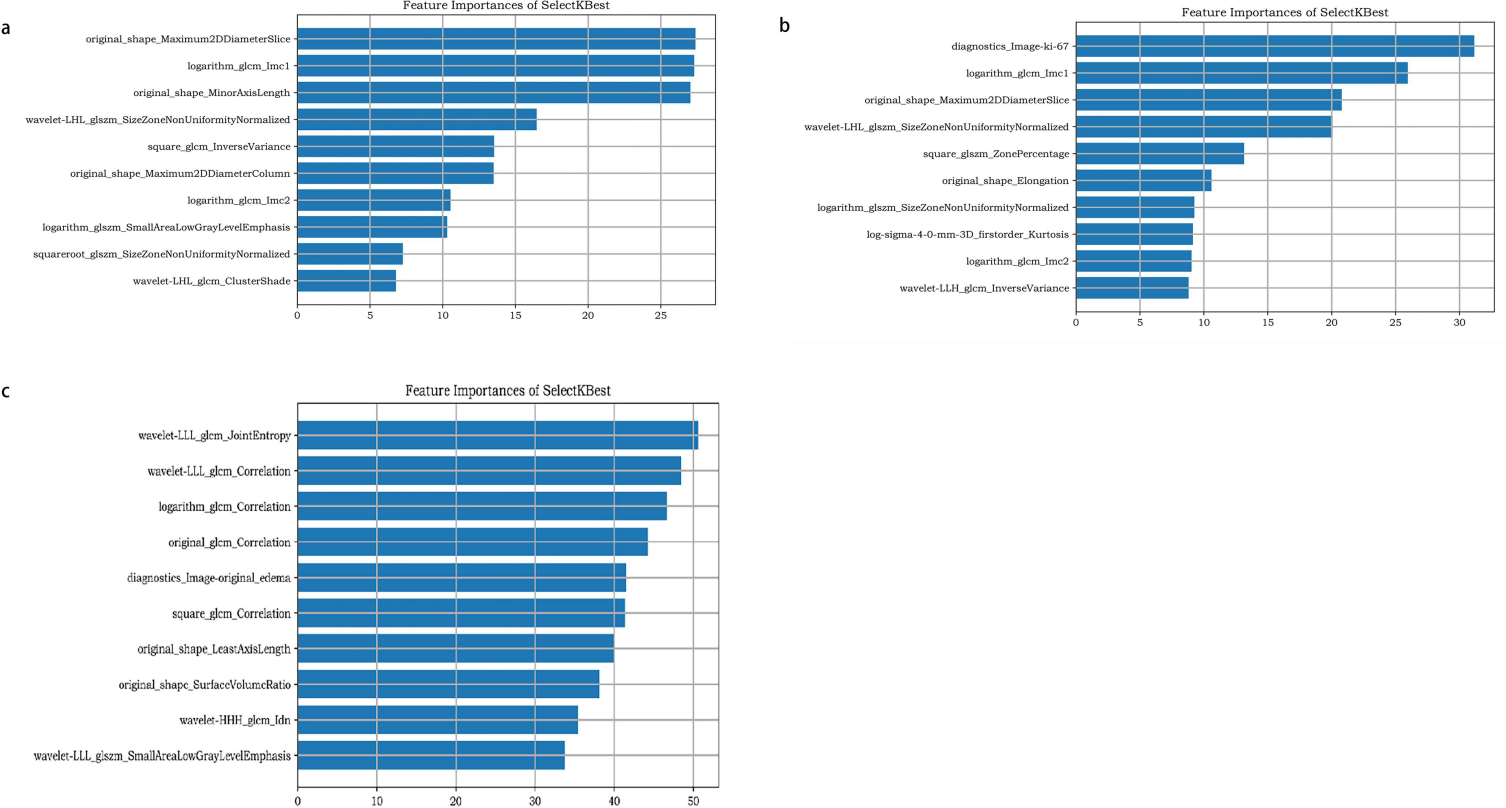
The ten most relevant features found of molecular prediction model **(a)**, recurrence prediction model, based on radiomics features **(b)**, and recurrence prediction model, based on radiomics features and clinical characteristics **(c)**.

The ten most relevant features found are demonstrated in **Figure 4**, including the Ki-67, GLCM, first-order morphological features, first-order statistical features, and their wavelet features.

### 3.4 Model prediction performance

**Figure 5:**
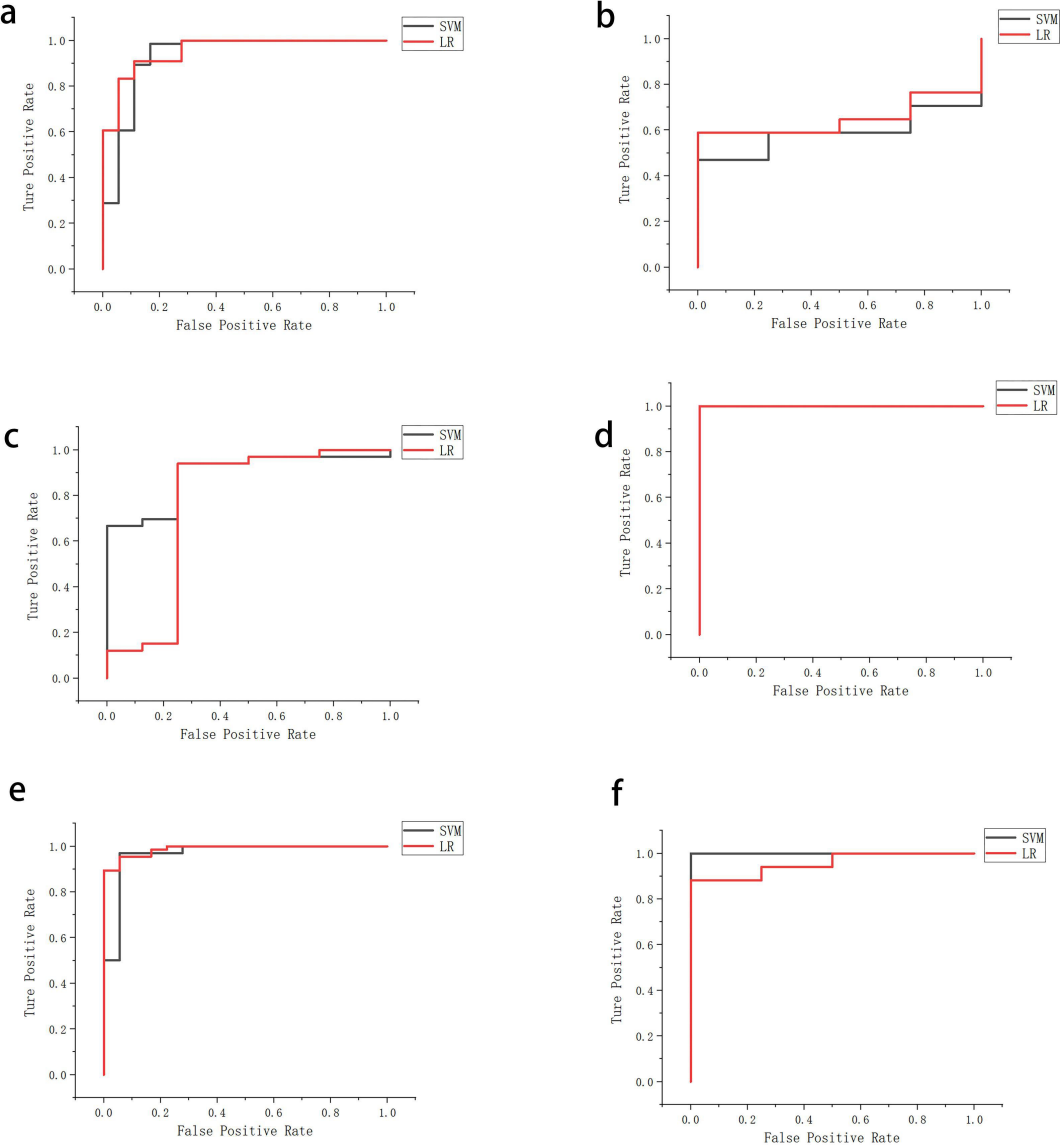
Performance of molecular prediction model **(a, b)**, recurrence prediction model, based on radiomics features **(c, d)**, and recurrence prediction model, based on radiomics features and clinical characteristics **(e, f)**, constructed based on SVM and LR in training set and test set.

**Figure 6:**
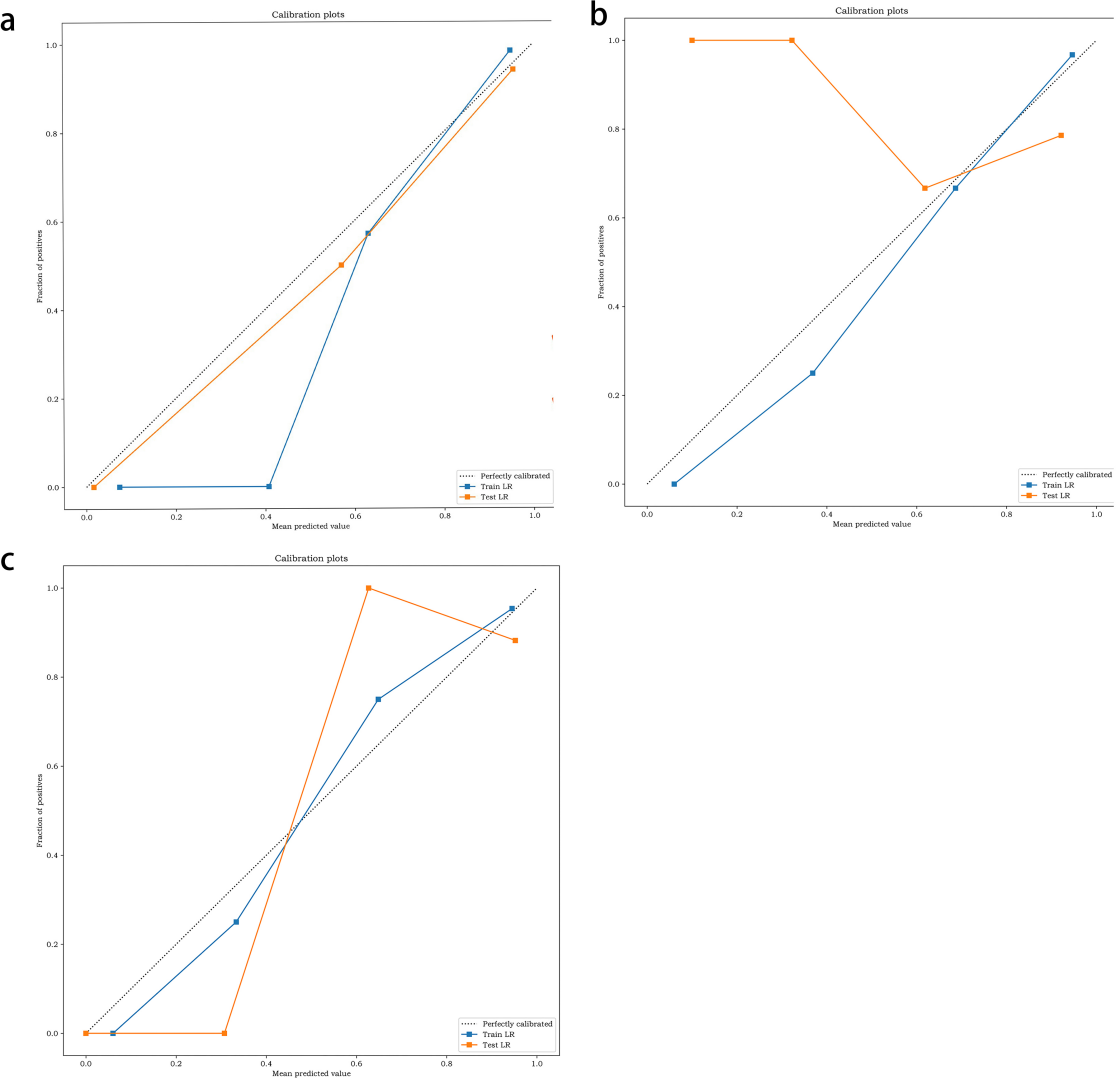
Calibration of molecular prediction model **(a)**, recurrence prediction model, based on radiomics features **(b)**, recurrence prediction model, based on radiomics features and clinical characteristics **(c)**.

**Figure 7:**
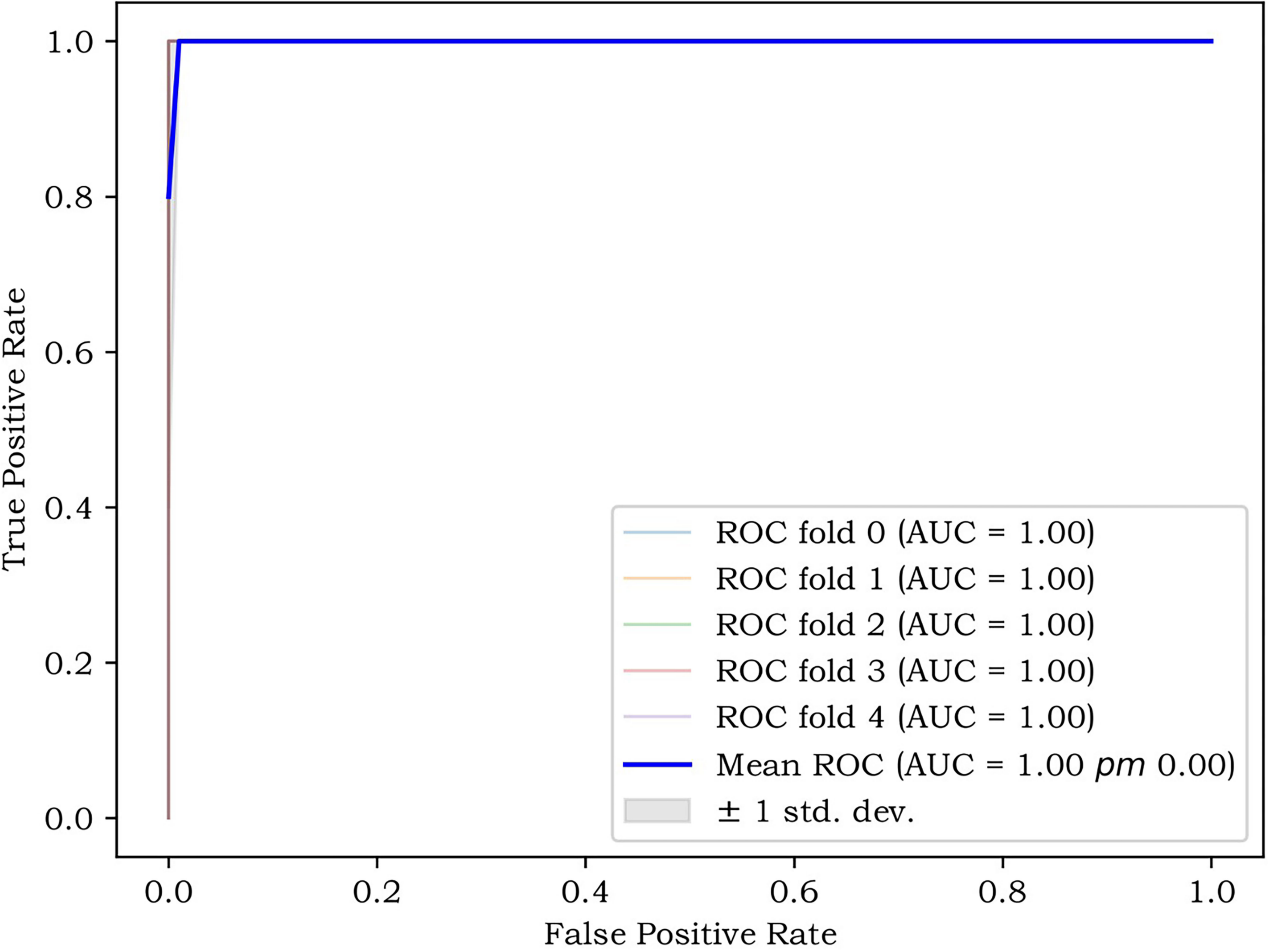
The K-fold cross-validation (CV), reflecting the stability of the model.

The performance of the two classifiers in different models is illustrated in **Figure 5**, respectively, and LR had the best performance. The results of the calibration curve and K-fold cross-validation are shown in **Figures 6** and **7**. The performances of the three models in predicting the molecular classification and recurrence of gliomas were as follows.

#### 3.4.1 Molecular prediction model

##### 3.4.1.1 SVM

The area under the curve (AUC) of the training set was 0.97, and, when the threshold was 0.82, the sensitivity, specificity, and accuracy of the set were 97%, 89%, and 97%, respectively (95% confidence interval [95%CI]: 0.91–1).

The AUC of the test set was 1, and, when the threshold was 0.67, the sensitivity, specificity, and accuracy of the set were 100%, 100%, and 100%, respectively (no 95%CI required).

##### 3.4.1.2 LR

The AUC of the training set was 0.99, and, when the threshold was 0.97, the sensitivity, specificity, and accuracy of the set were 95%, 88%, and 96%, respectively (95%CI: 0.97–1).

The AUC of the test set was 0.96, and, when the threshold was 0.88, the sensitivity, specificity, and accuracy of the set were 88%, 100%, and 100%, respectively (95%CI: 0.87–1).

#### 3.4.2 Recurrence prediction model, based on radiomics features

##### 3.4.2.1 SVM

The AUC of the training set was 0.89, and, when the threshold was 0.47, the sensitivity, specificity, and accuracy of the set were 93%, 75%, and 93%, respectively (95%CI: 0.78–1).

The AUC of the test set was 1, and, when the threshold was 0.83, the sensitivity, specificity, and accuracy of the set were 100%, 100%, and 100%, respectively (no 95%CI required).

##### 3.4.2.2 LR

The AUC of the training set was 0.76, and, when the threshold was 0.68, the sensitivity, specificity, and accuracy of the set were 94%, 75%, and 94%, respectively (95%CI: 0.5–1).

The AUC of the test set was 1, and, when the threshold was 0.81, the sensitivity, specificity, and accuracy of the set were 100%, 100%, and 100%, respectively (no 95%CI required).

#### 3.4.3 Based on radiomics features and clinical characteristics

##### 3.4.3.1 SVM

The AUC of the training set was 0.97, and, when the threshold was 0.82, the sensitivity, specificity, and accuracy of the set were 97%, 89%, and 97%, respectively (95%CI: 0.91–1).

The AUC of the test set was 1, and, when the threshold was 0.68, the sensitivity, specificity, and accuracy of the set were 100%, 100%, and 100%, respectively (no 95%CI required).

##### 3.4.3.2 LR

The AUC of the training set was 0.99, and, when the threshold was 0.67, the sensitivity, specificity, and accuracy of the set were 95%, 89%, and 96%, respectively (95%CI: 0.97–1).

The AUC of the test set was 0.96, and, when the threshold was 0.88, the sensitivity, specificity, and accuracy of the set were 88%, 100%, and 100%, respectively (95%CI: 0.87–1) (**Tables 1**, **2**).

**Table 1:** Performance of 2 models in the training set.

Models	Classifiers	AUC	95% CI	Specificity	Sensitivity	Accuracy
Model 1	SVM	0.97	0.91-1	0.97	0.89	0.97
	LR	0.99	0.97-1	0.95	0.88	0.96
Model 2	SVM	0.89	0.78-1	0.93	0.75	0.93
	LR	0.76	0.5-1	0.94	0.75	0.94
Model 3	SVM	0.97	0.91-1	0.97	0.89	0.97
	LR	0.99	0.97-1	0.95	0.89	0.96

**Table 2:** Performance of 2 models in the test set.

Models	Classifiers	AUC	95% CI	Specificity	Sensitivity	Accuracy
Model 1	SVM	1		1	1	1
	LR	0.96	0.97-1	0.88	1	1
Model 2	SVM	1		1	1	1
	LR	1		1	1	1
Model 3	SVM	1		1	1	1
	LR	0.96	0.87-1	0.88	1	1

## 4 Discussion

Gliomas are the most common malignant tumors of the central nervous system. Glioblastomas, due to their aggressiveness and rate of infiltration, have a high recurrence rate after surgery. Previous studies have reported that although there were interventions using secondary resection, radiotherapy, and chemotherapy to treat patients with recurrences, the results showed limited clinical benefit for patients (18). Therefore, early predictions of tumor recurrence, together with interventions and targeted, individualized treatments, are crucial for improving the prognoses of these patients.

Imaging techniques such as PET and MRI are highly valuable in predicting postoperative recurrence of gliomas, but there are many problems with these methods, including higher rates of false negativity, high prices, and lower accuracy. Taking amino acid PET as an example, although it can display the tumor metabolic activity area beyond the enhanced boundary detected by MRI through the biologically active volume (BTV), providing a more accurate range of infiltration for surgical planning (19). With the continuous development of artificial intelligence, the identification of microstructures and tumor microenvironments by radiomics provides a possibility for the early prediction of recurrences in patients with glioma using conventional imaging techniques. For example, the voxel-level radiomics model based on postoperative MRI (such as the CatBoost algorithm) has demonstrated good performance in an external cohort, with an average AUC of 0.81 and an accuracy of 84%. Moreover, the predicted recurrence area is highly spatially consistent with the actual recurrence (20).

In this study, the radiomics features were extracted from conventional MRI, and a predictive model was developed for an early prediction of the molecular classification and postoperative recurrence in patients with glioma. The results indicated that a comprehensive model combining the clinical features and radiomics features could better assist in diagnosing the postoperative recurrence of gliomas. As an independent predictor of the prognosis in glioma, the IDH level is generally considered more objective and reliable than current clinical criteria (21). However, in this study, it was suggested that the Ki-67 level was a factor more relevant to the recurrence of gliomas. The reconstructed model, which removed the irrelevant features, including IDH expression, but incorporated highly relevant features such as the Ki-67 level, was reconstructed, and it was revealed that the final model had a better performance, which further verified the high correlation between Ki-67 and the recurrence of gliomas. However, owing to the small sample size of this study, there might be some bias.

The ten radiomic features screened could be roughly divided into four categories: (1) first-order morphological features, (2) Ki-67 as the clinical feature, (3) GLCM and GLSZM as the texture features, and (4) the features with the conduction of wavelet transformation on the first several feature. The high recurrence of Ki-67 indicated that tumor recurrence was correlated with the abnormal proliferation of tumor cells. Because the spatial distribution of grayscale is adopted in the GLCM to characterize texture, the spatial distributions of textures with different thicknesses are different. Their high recurrence also indicated that the recurrence of gliomas might be closely correlated with spatial heterogeneity. To verify the correlation, the model was not only reconstructed using these features and not only performed 5-fold cross-validation but also verified the correlation between these features extracted from a single sequence and glioma recurrence in this study. The correlated features, extracted by the T1WI and T2WI/FLAIR images, failed to predict the recurrence of gliomas successfully, except for the CE-T1 sequence. Tumor tissues showed similar signals on the T1WI, which might be why features extracted from the T1WI sequence alone failed to predict the recurrence of gliomas successfully. The prediction model was developed by combining the CE-T1 sequence and radiomics score (Radcore), including the T1WI and T2WI/FLAIR features, and the results revealed a strong prediction performance. This suggested that multi-feature analysis along with the Radscore might be more effective than traditional texture features in predicting the recurrence of gliomas. And this result was consistent with what has been demonstrated in previous studies, that is, the TI value can distinguish tumor infiltration from edema, a high TI value corresponds to the area with a high recurrence risk at the edge of the surgical cavity, and it is related to the preservation of neurological function after surgery (22). However, the biological significance of some of the radiomics features in this study still remains unknown, and further research is needed to investigate the potential significance in the future.

In this study, as a relatively traditional and robust nonlinear classifier, the SVM showed poor prediction performance on the test set, which was inconsistent with previous studies. On the one hand, it might be related to the small sample size of the test set. On the other hand, as a classic linear classifier, whether the uniformly better prediction performance of the LR in the training and test sets could indicate that the prediction of glioma recurrences might be more related to the extraction and screening of linear features was a question that had not been addressed in previous studies.

Previous studies primarily focused on adopting radiomics methods to distinguish whether a glioma was benign or malignant and evaluate the postoperative efficacy. Some studies did not consider clinical factors. However, these studies mainly focused on the application of radiomics for the therapeutic evaluation of recurrent malignant gliomas, and most studies did not consider the clinical factors (23, 24). The significance of this study lies in the early prediction of glioma recurrences. By extracting the features of recurrence areas at different time points multiple times, we screen out features related to recurrence and establish a prediction model to achieve early prediction of glioma recurrence and high-recurrence general areas.

The comprehensive model showed the ability to predict early the recurrence of gliomas and predilection sites in both the training and test sets. To eliminate the selection bias caused by the small sample size as much as possible, the K-fold cross-validation method was adopted, and the features were re-screened through multiple cross-validations; the results proved that the model had good stability. The results of the statistical analysis also revealed that the statistical difference between the two cohorts was not significant. Therefore, building a multiparametric radiomics model was a feasible solution for the early prediction of glioma recurrences, which was also in line with the current trend of individualized medicine (17, 25).

This study also attempted to incorporate the macroscopic radiographical features (tumor location, midline spanning, and degree of peri-tumor edema) into the comprehensive model, but the results showed no high recurrence indicators, which indicated that these features did not significantly correlate with the recurrence of gliomas. The texture features were extracted from the T1WI and T2/FLAIR images through a filtering step, with extraction and enhancement of the features of different sizes corresponding to fine, medium, and coarse texture scales. Because of these features, the constructed model better predicted postoperative recurrence of gliomas with a strong performance, demonstrating that texture analysis might predict the postoperative recurrence of gliomas by quantifying heterogeneity without additional unconventional imaging.

The image segmentation method is a very important factor for radiomics. In recent years, automatic segmentation has become increasingly popular among researchers due to the development of deep learning. This study adopted a combination of manual and semi-automatic segmentation methods. Although different from the mainstream methods, manual segmentation had the advantage of enabling the doctors to modify the ROI based on their own recognition since there was information that was difficult to be explained by the current automatic segmentation technology in the images. Moreover, the final performance of the radiomics model not only depended on the image segmentation method but also on the selection of clinical information, the degree of differentiation of the tumor and other factors.

There were certain limitations in this study. First, this was a retrospective study, and there might have been selection bias. Second, most of the subjects included in the present study were those with tumors classified as grades III to IV by WHO. If those with low-grade gliomas could be enrolled in the study, comparing recurrences between different grades could make the correlation between the recurrence of gliomas and the spatial heterogeneity more convincing. In addition, the sample size was small and lacked external validation. There was the possibility of model over-fitting, which should be solved through continuous optimization of dimensionality reduction methods. The model in this study uses the default parameters of sklearn library without tuning parameters, which might also contribute to sub-optimal performance. In the future, it is also advisable to consider incorporating the characterization of transcriptional heterogeneity by radio genomic features, integrating more molecular markers (such as factors related to the circ0030018/miR-1236/HER2 pathway and functional imaging parameters, so as to further optimize the universality and predictive efficacy of the model (3, 16).

In conclusion, it was demonstrated in this study that combining the MRI-based multiparametric radiomic model with clinical features, molecular information, and basic imaging features might have complementary effects and could improve the early predictions of postoperative glioma recurrences and the areas of high recurrence with a strong performance. In the future, the investigation will focus on integrating more molecular markers, radiomics signatures, and functional imaging to apply clinical decision support tools for individualized treatment decisions and improve the patients’ quality of life.

## Data Availability

The raw data supporting the conclusions of this article will be made available by the authors, without undue reservation.
